# Repeated Pancreatectomy for Isolated Local Recurrence in the Remnant Pancreas Following Radical Pancreatectomy for Pancreatic Ductal Adenocarcinoma: A Pooled Analysis

**DOI:** 10.3390/jcm9123945

**Published:** 2020-12-05

**Authors:** Munseok Choi, Na Won Kim, Ho Kyoung Hwang, Woo Jung Lee, Chang Moo Kang

**Affiliations:** 1 Division of Hepatobiliary and Pancreatic Surgery, Department of Surgery, Yonsei University College of Medicine, Seoul 03722, Korea; cms2598@yuhs.ac (M.C.); DRHHK@yuhs.ac (H.K.H.); wjlee@yuhs.ac (W.J.L.); 2 Yonsei University of Medical Library, Seoul 03722, Korea; NWKIM@yuhs.ac

**Keywords:** completion total pancreatectomy, pancreatic ductal adenocarcinoma, pooled analysis, recurrent pancreatic cancer, repeated pancreatectomy, survival

## Abstract

The mainstream treatment for recurrent pancreatic cancer is potent chemotherapy or chemoradiotherapy. However, recent clinical investigations have suggested a potential oncologic role of local resection of recurrent pancreatic cancer. This systemic review with a pooled analysis aimed to assess the potential role of local repeated pancreatectomy with respect to the survival outcomes for patients with recurrent pancreatic ductal adenocarcinoma (PDAC) in the remnant pancreas. The PubMed database was searched, and 15 articles reporting on repeated pancreatectomy for local recurrence of PDAC in the remnant pancreas were identified. The pooled individual data were examined for the clinical outcomes of repeated pancreatectomy for recurrent PDAC. The survival analysis was performed using the Kaplan–Meier method. In the pooled analysis, the mean time interval from initial pancreatectomy to repeated pancreatectomy was 41.3 months (standard deviation (SD), 29.09 months). Completion total pancreatectomy was most commonly performed as repeated pancreatectomy (46 patients, 92.0%), and partial pancreatic resection was performed for only 4 (10.3%) patients. Twenty (40.9%) patients received postoperative chemotherapy following repeated pancreatectomy. The median overall survival was 60 months (95% confidential interval (CI): 45.99–74.01) after repeated pancreatectomy for isolated local recurrence in the remnant pancreas. Overall survival was markedly longer considering the timing of the initial pancreatectomy for pancreatic cancer (median, 107 months (95% CI: 80.37–133.62). The time interval between the initial and subsequent repeated pancreatectomy for pancreatic cancer was not associated with long-term oncologic outcomes (*p* = 0.254). Repeated pancreatectomy cannot completely replace adjuvant chemotherapy but should be considered for patients with isolated local recurrent PDAC in the remnant pancreas.

## 1. Introduction

Despite low resection rates at the initial diagnostic stage, margin-negative resection is the only strategy to ensure long-term survival when treating patients with pancreatic cancer. However, recurrence is high in patients with resected pancreatic cancer. Up to 80% of patients who undergo curative pancreatectomy will experience systemic or local recurrence within 2 years [1]. According to the available literature, isolated local recurrence without systemic metastasis is reported in up to 30% of patients [2,3].

International consensus concerning the role of surgical management for patients with isolated local recurrence of pancreatic ductal adenocarcinoma (PDAC) in the remnant pancreas has not been achieved. Importantly, an adequate number of cases of treatment for isolated local recurrence of PDAC in the remnant pancreas have not been documented; therefore, the treatment required for recurrent PDAC has not been discussed in detail. The mainstream treatment for isolated local recurrence of PDAC in the remnant pancreas was potent chemotherapy or chemoradiotherapy. However, recent clinical investigations have suggested a potential oncologic role of local resection of recurrent pancreatic cancer [4,5,6]. This study aimed to evaluate the potential role of repeated pancreatectomy for isolated local recurrence of PDAC in the remnant pancreas using a pooled analysis, and to scrutinize the oncologic significance of the reported studies on repeated pancreatectomy so far.

## 2. Materials and Methods

### 2.1. Search Strategy and Data Sources

An extensive literature review was conducted according to the 2009 Preferred Reporting Items for Systematic Reviews and Meta-Analyses (PRISMA) guidelines [7]. The PubMed (MEDLINE) database was searched for articles published between January 2000 and April 2020 using the following terms: remnant pancreatic cancer, pancreatic cancer, pancreatectomy, pancreatic resection, local neoplasm recurrence, completion pancreatectomy, remnant pancreas, pancreatic ductal adenocarcinoma, recurrent pancreatic cancer, and second pancreatectomy. The variables of interest included sex, age, surgical procedures, disease-free interval between the initial pancreatectomy and appearance of remnant PDAC, R status (Resection margin status), adjuvant chemotherapy, 30-day mortality, and overall survival (OS). Isolated local recurrence of PDAC was defined as first recurrence limited to the remnant pancreas.

### 2.2. Inclusion and Exclusion Criteria

The inclusion criteria were (1) repeated pancreatectomy for isolated locally recurrent pancreatic cancer limited to the remaining pancreas after pancreatic resection, (2) evaluation of at least one of the clinicopathological or survival characteristics, (3) published original articles or case reports that contained original data, and (4) cases with pathologically confirmed ductal adenocarcinoma and with data available on both individualized long-term survival and time interval. All studies that did not meet the inclusion criteria were excluded. In addition, the following exclusion criteria were applied: (1) absence of data for individual patients, (2) other types of pancreatic cancer except pancreatic ductal adenocarcinoma, and (3) written in languages other than English. Two independent reviewers (MSC and CMK) reviewed all the retrieved studies that met the inclusion and exclusion criteria by manually screening the articles. Discrepancies between the two reviewers were resolved by discussion and achieving a team consensus.

### 2.3. Statistical Analysis

All statistical analyses were performed using SPSS Statistical software (version 25.0; SPSS Inc., Chicago, IL, USA). The continuous variables were expressed as means ± standard deviations or ranges, and the categorical variables were expressed as frequencies or percentages. The Student’s t-test was used to compare the continuous variables, and the chi-square tests and Fisher’s exact tests were used to compare the categorical data. The Kaplan–Meier method was used for the analysis of the OS. To identify the potential factors predicting the OS, univariate and multivariate analyses of the clinicopathological variables were performed using Cox-proportional hazard regression models with backward elimination. A *p*-value < 0.05 was considered statistically significant.

## 3. Results

### 3.1. General Characteristics of the Patients

**Figure 1:**
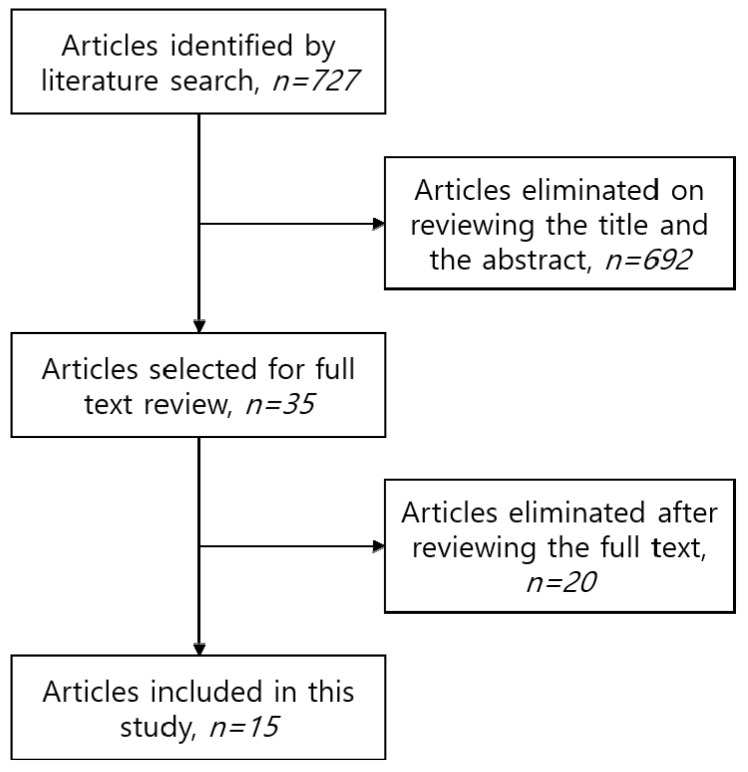
Flow chart for study selection.

A total of 727 potential studies were identified. Overall, 692 studies were excluded on reviewing the title and abstract. Thirty-five studies were selected for full-text review [3,4,6,8,9,10,11,12,13,14,15,16,17,18,19,20,21,22,23,24,25,26,27,28,29,30,31,32,33,34,35,36,37,38]. Of these, 15 studies met the inclusion criteria and were included in our pooled analysis [8,11,12,13,14,15,17,21,23,24,25,29,30,31,39] (Figure 1). The 15 studies are summarized in Table 1.

The overall incidence of isolated local recurrence in the remnant pancreas was reported in 8 of 15 articles and showed a distribution of 0.3–5.3%. Among 50 patients, 18 male and 32 female patients with a mean age of 65.0 (range 57.15–72.85) years were identified. Pancreaticoduodenectomy was performed for 32 (64.0%) patients, distal pancreatectomy for 17 (34.0%) patients, and partial pancreatic resection for 1 (2.0%) patient as the initial pancreatectomy for pancreatic cancer. The mean time interval from initial pancreatectomy to repeated pancreatectomy was 41.3 (range 12.21–70.39) months. Completion total pancreatectomy was most commonly performed as repeated pancreatectomy (46 patients, 92.0%), and partial pancreatic resection was performed for only 4 (10.3%) patients. Twenty (40.9%; missing data, 17 patients, 34.0%) patients received postoperative chemotherapy following repeated pancreatectomy (Table 2).

### 3.2. Long-Term Oncologic Outcomes

**Figure 2:**
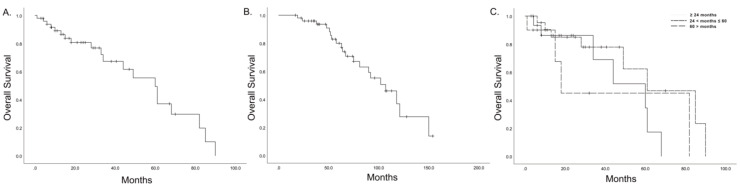
(**A**) Overall survival after repeated pancreatectomy. (**B**) Overall survival calculated from the time of the initial pancreatectomy. (**C**) Overall survival according to the time interval from the initial radical pancreatectomy.

The median OS was 60 (95% confidential interval (CI): 45.9–74.0) months after repeated pancreatectomy for isolated local recurrence in the remnant pancreas (Figure 2A). The median OS was markedly longer if the follow-up duration was calculated from the time of the initial pancreatectomy (107 months, 95% CI: 80.3–133.0, Figure 2B). The time interval between the initial and repeated pancreatectomy was not associated with the long-term oncologic outcome of repeated pancreatectomy (*p* = 0.254; Figure 2C). In univariate analysis, the time interval between the initial and repeated pancreatectomy, R1 resection, and adjuvant chemotherapy were not associated with the OS after repeated pancreatectomy (Table 3).

### 3.3. Short-Term Operative Outcomes

Of those 50 patients, none experienced 30-day mortality, and only one patient died within 90 days after repeated pancreatectomy. Postoperative complications mentioned explicitly in the literature were delayed gastric emptying (*n* = 2), intra-abdominal abscess (*n* = 4), sepsis (*n* = 1), focal hepatic infarction (*n* = 1), and subcutaneous abscess (*n* = 1). Among all 50 cases, 11 did not mention postoperative complications.

## 4. Discussion

In this study, we aimed to evaluate the potential role of local repeated pancreatectomy for recurrent PDAC in the remnant pancreas, and we found that repeated pancreatectomy improved the survival outcomes for patients with isolated local recurrent PDAC in the remnant pancreas. Pancreatic cancer is considered one of the dismal malignant diseases in the gastrointestinal system. Only margin-negative resection is essential for long-term survival; however, the resection rate at diagnostic stage is low, and recurrence is commonly noted within 2 years, even after radical pancreatectomy [40]. Finally, disease progression leading to cancer-related mortality during chemotherapy is inevitable in patients with recurrence. However, this clinical scenario may not always hold true owing to the recent changes in clinical oncology, such as the development of advanced surgical techniques, perioperative management strategies, and improved potent chemotherapeutic agents.

Unlike systemic recurrence, isolated local recurrence of pancreatic cancer is considered a topic of interest for pancreatic surgeons because recurrence can be controlled by local treatment, such as repeated pancreatectomy, in selected patients. Especially, considering the potential role of neoadjuvant chemotherapy in treating pancreatic cancer, chemotherapy for local recurrence in the remnant pancreas followed by repeated pancreatectomy may potentially be an option for treating isolated local recurrent pancreatic cancer [41]. Neoadjuvant chemotherapy is used for treating isolated recurrence of pancreatic cancer in the remnant pancreas with the aim of treating possible microscopic systemic metastasis that cannot be detected, and assessing tumor biology for selecting the appropriate patients.

With respect to the long-term oncologic outcomes of repeated pancreatectomy for isolated local recurrence of pancreatic cancer, Yamada, et al. reported 114 patients with remnant pancreatic cancer after initial pancreatectomy [6]. Ninety patients underwent repeated pancreatectomy; the median survival was 26 months, which was superior to that noted for patients who did not undergo resection (hazard ratio (HR): 0.56, *p* = 0.012). Hashimoto et al. reviewed 12 published studies reporting on recurrent pancreatic cancer in the remnant pancreas following initial pancreatectomy, and they showed that the OS after repeated pancreatectomy for remnant pancreatic cancer was 14–35.5 months, which was markedly longer than that noted for patients with unresectable pancreatic cancer in recent studies [24]. Groot et al. performed a systemic review of the treatment of isolated local recurrence of pancreatic cancer. Based on eight published studies including 100 patients who underwent re-resection of recurrent pancreatic cancer, they concluded that local re-resection of recurrent pancreatic cancer may be feasible, safe, and effective in the selected patients [33]. They demonstrated that the postoperative morbidity and mortality rates were 29% and 1%, respectively. In addition, the median survival was markedly higher (32 months) compared to that for other treatment modalities, such as chemotherapy (19 months) and radiotherapy (16 months). Zhou et al. reported that repeated pancreatectomy can be safely performed in recurrent PDAC and showed good long-term results by conducting a literature review from 2000 to 2016 with pooled analysis, which is the same analysis method as the present study [32]. The present study was conducted for a literature review by adding case reports or case series for the extended period.

The reason why patients with isolated local recurrence of pancreatic cancer have better prognosis than those with other sites of distant dissemination of the disease is a matter of debate. What is the reasonable basis for better survival in isolated local recurrence of pancreatic cancer? At first, taking into account the biological background of isolated local recurrence patients, obtaining a survival benefit through surgical treatment could be a well-founded treatment strategy. Research has shown that pancreatic cancer is likely to be exposed to distant metastasis prior to surgical resection [42]. In an autopsy series, Haeno et al. revealed that a small subset of patients died with only locally advanced disease, suggesting that some tumors may lack metastasis-promoting factors (or have metastasis-suppressing factors) or may have metastases that are especially sensitive to systemic chemotherapy [43]. This is thought to be directly related to the high median OS highlighted in the present study. Furthermore, the role of adjuvant therapy is also significant. In the ESPAC-4 trial, the patient group using the combination of gemcitabine with capecitabine showed a better DFS and OS than the group of patients using gemcitabine alone [44]. This result should be considered for one factor that improves survival. Although further study is necessary, the first surgery dissects the soft tissue (nerve, lymphatics) and blocks the route to propagate the tumor to the surrounding area. Besides, most patients recur in a highly attenuated state of potential residual cancer cells by adjuvant chemotherapy after the first surgery. Therefore, there is a possibility that it remains purely isolated recurrence, and there is room for improvement in oncologic outcome through repeated pancreatectomy.

Operating on a recurrent PDAC in the remnant pancreas is challenging as the procedure may be associated with high morbidity or mortality due to adhesion of the tumor with the surrounding tissue and anatomical deformation after the surgery. However, according to recent reports, repeated pancreatectomy is safe [4]. According to our limited experiences, completion total pancreatectomy for isolated recurrence in the remnant pancreas after initial pancreaticoduodenectomy is technically demanding as a safe surgical procedure, especially when the previous pancreatic division line is above the Superior mesenteric vein-splenic vein-portal vein confluence. Pancreaticojejunostomy associated postoperative pancreatic fistula (POPF) may result in severe adhesion around these venous vascular systems and the celiac axis where the remnant distal pancreas and these major vascular structures should be dissected safely; thus, difficulties are encountered in performing repeated pancreatectomy. Therefore, as Fortner suggested, the pancreatic division may be performed distal to the splenic artery origin during the initial pancreaticoduodenectomy for resectable pancreatic cancer, considering the possibility of subsequent repeated pancreatectomy for isolated recurrent pancreatic cancer in the remnant pancreas [45].

Although long-term follow-up is required to address the potential role of repeated pancreatectomy for recurrent pancreatic cancer, recent studies and the present pooled analysis strongly suggest the oncologic benefits of this approach [36,37]. In the present study, the median OS was estimated to be 60 months from the time of repeated pancreatectomy and 107 months from the time of initial radical pancreatectomy. Although the R1 resection rate after repeated pancreatectomy was higher than that noted after the first pancreatectomy (8.5% vs. 15.5%, Table 2), the R1 resection rate was not associated with the OS after pancreatectomy in univariate analysis (Table 3). Therefore, repeated pancreatectomy is a challenging procedure, but preparing for R1 resection and attempting surgical treatment may benefit the patient with respect to the OS.

In addition, it is quite difficult to differentiate between local recurrence and de novo carcinogenesis, especially when the duration from the initial pancreatectomy is quite long. However, regardless of the duration, the present pooled analysis showed that there was no difference with respect to survival after repeated pancreatectomy was successfully performed. Therefore, medical oncologists and pancreatic surgeons should consider that the patients with isolated local recurrence in the remnant pancreas following initial radical pancreatectomy may not show poor prognosis but may be able to survive long-term if repeated pancreatectomy can be safely performed.

When reviewing the literature, it was found that isolated local recurrence is rare compared to systemic recurrence following radical pancreatectomy for pancreatic cancer. Therefore, it is thought that very selected cases were collected and analyzed, resulting in difficulty for generalization. However, despite a rare recurrent pattern of resected pancreatic cancer, these patients are potentially encountered in clinical practice. In addition, with the advance of laparoscopic technique, even laparoscopic repeated pancreatectomy seems to be feasible in recurred pancreatic cancer [46]. What shall we do for them? The present analysis may not be generalized but can at least provide potential treatment options for the patients. Further experiences and investigations are mandatory to reveal the potential oncologic role of local resection in isolated local recurrence of resected pancreatic cancer.

A limitation of the present study is that potential clinically important factors, such as tumor differentiation, lymph node metastasis, and perineural invasion, were not considered in this analysis. A more reliable analysis would have been possible if the original data of the cohort study, including a relatively larger number of cases specifying clinic-pathological variables, could be obtained. In the near future, a risk model for predicting isolated local recurrence following initial pancreatectomy for pancreatic cancer should be developed based on data from a well-designed multicenter collaborative study.

In conclusion, we revealed that repeated pancreatectomy for isolated local recurrence in the remnant pancreas could improve survival outcomes based on the pooled analysis of data from published studies. Hence, a case-specific surgical approach for repeated pancreatectomy for recurrent pancreatic cancer, such as indications, the extent of surgery, and prognostic factors, should be established based on consensus and more reliable, convincing data.

## 5. Conclusions

Repeated pancreatectomy cannot completely replace the role of adjuvant chemotherapy but should be considered for patients with isolated local recurrent PDAC in the remnant pancreas.

## Figures and Tables

**Table 1:** Characteristics of the selected studies.

Author, Year	Study Design	Incidence ^†^, %	*n*	1st Operation	Time Interval (Months, Mean)	2nd Operation	OS (Months, Median)
				PD/DP/PP		CTP/PP	
Eriguchi, 2000 [8]	Case report	NA	1	0/1/0	88.0	1/0	8.0
Takamatsu, 2005 [11]	Case report	NA	1	1/0/0	47.0	1/0	10.0
Dalla Valle, 2006 [12]	Case report	NA	1	1/0/0	15.0	1/0	24.0
Miura, 2007 [13]	Case series, single center	2.3	1	1/0/0	20.0	1/0	44.0
Tajima, 2008 [14]	Case report	NA	1	1/0/0	37.0	1/0	38.0
Koizumi, 2010 [15]	Case report	NA	2	1/1/0	59.5	2/0	9.0
Lavu, 2011 [17]	Case series, single center	1.3	3	2/1/0	68.0	3/0	8.0
Shimoike, 2013 [21]	Case report	NA	2	1/1/0	18.0	2/0	24.0
Boone, 2013 [23]	Case series, single center	NA	6	6/0/0	29.9	4/2	31.0
Hashimoto, 2014 [24]	Case series, single center	2.6	6	3/3	29.7	6/0	15.5
Miyazaki, 2014 [25]	Case series, single center	5.3	9	6/3/0	32.7	8/1	28.0
Shima, 2015 [39]	Case series, single center	3.2	6	4/2/0	28.8	5/1	27.5
Ishida, 2016 [29]	Case series, single center	0.8	1	0/1/0	53.0	1/0	21.0
Sahakyan, 2016 [30]	Case report	0.3	1	1/0/0	36.0	1/0	4.0
Suzuki, 2016 [31]	Case series, single center	1.1	9	4/4/1	64.7	9/0	15.0

PD, pancreaticoduodenectomy; DP, distal pancreatectomy with or without splenectomy; CTP, completion total pancreatectomy; PP, partial pancreatectomy. ^†^ Incidence, isolated local recurrence in the remnant pancreas/total resected pancreatic cancer.

**Table 2:** Characteristics of the patients undergoing repeated pancreatectomy for recurrent pancreatic ductal adenocarcinoma included in the pooled analysis.

	*n* = 50
Age	65.0 ± 7.85
Sex, male	18 (40.9)
Type of 1st OP	
PD	32 (64.0)
DP	17 (34.0)
TP	0 (0.0)
PP	1 (2.0)
Combined resection	11 (22.0)
R status, 1st OP	
R0	43 (91.5)
R1 or R2	4 (8.5)
Adjuvant CTx., 1st OP	25 (58.1)
Time interval	41.3 ± 29.09
Type of 2nd OP	
CTP	46 (92.0)
PP	4 (10.3)
R status, 2nd OP	
R0	32 (84.2)
R1 or R2	6 (15.8)
30-day mortality	0 (0.0)
90-day mortality	1 (2.0)
Adjuvant CTx., 2nd OP	20 (40.9)

OP, operation; PD, pancreaticoduodenectomy; DP, distal pancreatectomy with or without splenectomy; TP, total pancreatectomy; PP, partial pancreatectomy; CTx, chemotherapy; CTP, completion total pancreatectomy.

**Table 3:** Univariate analysis of the predictors of overall survival after repeated pancreatectomy.

Variables	HR	95% CI	*p*-Value
Time interval			
≤24 months			0.280
24 < months ≤ 60	0.460	0.152–1.390	0.169
>60 months	1.096	0.308–3.907	0.887
R1 resection, repeated pancreatectomy	2.785	0.287–27.007	0.377
Adjuvant CTx after repeated pancreatectomy	3.704	0.788–17.418	0.097

CTx, chemotherapy; CI, confidence interval.

## References

[1] Garcea G., Dennison A.R., Pattenden C.J., Neal C.P., Sutton C.D., Berry D.P. (2008). Survival following curative resection for pancreatic ductal adenocarcinoma. A systematic review of the literature. Jop.

[2] Van den Broeck A., Sergeant G., Ectors N., Van Steenbergen W., Aerts R., Topal B. (2009). Patterns of recurrence after curative resection of pancreatic ductal adenocarcinoma. Eur. J. Surg. Oncol..

[3] Hishinuma S., Ogata Y., Tomikawa M., Ozawa I., Hirabayashi K., Igarashi S. (2006). Patterns of recurrence after curative resection of pancreatic cancer, based on autopsy findings. J. Gastrointest. Surg..

[4] Hashimoto D., Arima K., Nakagawa S., Negoro Y., Hirata T., Hirota M., Inomata M., Fukuzawa K., Ohga T., Saeki H. (2019). Pancreatic cancer arising from the remnant pancreas after pancreatectomy: A multicenter retrospective study by the kyushu study group of clinical cancer. J. Gastroenterol..

[5] Groot V.P., Rezaee N., Wu W., Cameron J.L., Fishman E.K., Hruban R.H., Weiss M.J., Zheng L., Wolfgang C.L., He J. (2018). Patterns, timing, and predictors of recurrence following pancreatectomy for pancreatic ductal adenocarcinoma. Ann. Surg..

[6] Yamada S., Kobayashi A., Nakamori S., Baba H., Yamamoto M., Yamaue H., Fujii T. (2018). Resection for recurrent pancreatic cancer in the remnant pancreas after pancreatectomy is clinically promising: Results of a project study for pancreatic surgery by the japanese society of hepato-biliary-pancreatic surgery. Surgery.

[7] Moher D., Liberati A., Tetzlaff J., Altman D.G. (2009). Preferred reporting items for systematic reviews and meta-analyses: The prisma statement. PLoS Med..

[8] Eriguchi N., Aoyagi S., Imayama H., Okuda K., Hara M., Fukuda S., Tamaie T., Kanazawa N., Noritomi T., Hiraki M. (2000). Resectable carcinoma of the pancreatic head developing 7 years and 4 months after distal pancreatectomy for carcinoma of the pancreatic tail. J. Hepato-Biliary-Pancreat. Surg..

[9] Wada K., Takada T., Yasuda H., Amano H., Yoshida M. (2001). A repeated pancreatectomy in the remnant pancreas 22 months after pylorus-preserving pancreatoduodenectomy for pancreatic adenocarcinoma. J. Hepato-Biliary-Pancreat. Surg..

[10] Doi R., Ikeda H., Kobayashi H., Kogire M., Imamura M. (2003). Carcinoma in the remnant pancreas after distal pancreatectomy for carcinoma. Eur. J. Surg. Suppl..

[11] Takamatsu S., Ban D., Irie T., Noguchi N., Kudoh A., Nakamura N., Kawamura T., Igari T., Teramoto K., Arii S. (2005). Resection of a cancer developing in the remnant pancreas after a pancreaticoduodenectomy for pancreas head cancer. J. Gastrointest. Surg..

[12] Dalla Valle R., Mancini C., Crafa P., Passalacqua R. (2006). Pancreatic carcinoma recurrence in the remnant pancreas after a pancreaticoduodenectomy. J. Pancreas.

[13] Miura F., Takada T., Amano H., Yoshida M., Isaka T., Toyota N., Wada K., Takagi K., Kato K. (2007). Repeated pancreatectomy after pancreatoduodenectomy. J. Gastrointest. Surg..

[14] Tajima Y., Kuroki T., Ohno T., Furui J., Tsuneoka N., Adachi T., Mishima T., Kosaka T., Haraguchi M., Kanematsu T. (2008). Resectable carcinoma developing in the remnant pancreas 3 years after pylorus-preserving pancreaticoduodenectomy for invasive ductal carcinoma of the pancreas. Pancreas.

[15] Koizumi M., Sata N., Kasahara N., Morishima K., Sasanuma H., Sakuma Y., Shimizu A., Hyodo M., Yasuda Y. (2010). Remnant pancreatectomy for recurrent or metachronous pancreatic carcinoma detected by fdg-pet: Two case reports. J. Pancreas.

[16] Kinoshita H., Yamade N., Nakai H., Sasaya T., Matsumura S., Kimura A., Shima K. (2011). Successful resection of pancreatic carcinoma recurrence in the remnant pancreas after a pancreaticoduodenectomy. Hepatogastroenterology.

[17] Lavu H., Nowcid L.J., Klinge M.J., Mahendraraj K., Grenda D.R., Sauter P.K., Rosato E.L., Kennedy E.P., Yeo C.J. (2011). Reoperative completion pancreatectomy for suspected malignant disease of the pancreas. J. Surg. Res..

[18] Katz M.H., Wang H., Balachandran A., Bhosale P., Crane C.H., Wang X., Pisters P.W., Lee J.E., Vauthey J.N., Abdalla E.K. (2012). Effect of neoadjuvant chemoradiation and surgical technique on recurrence of localized pancreatic cancer. J. Gastrointest. Surg..

[19] Kobayashi T., Sato Y., Hirukawa H., Soeno M., Shimoda T., Matsuoka H., Kobayashi Y., Tada T., Hatakeyama K. (2012). Total pancreatectomy combined with partial pancreas autotransplantation for recurrent pancreatic cancer: A case report. Transplant. Proc..

[20] Thomas R.M., Truty M.J., Nogueras-Gonzalez G.M., Fleming J.B., Vauthey J.N., Pisters P.W., Lee J.E., Rice D.C., Hofstetter W.L., Wolff R.A. (2012). Selective reoperation for locally recurrent or metastatic pancreatic ductal adenocarcinoma following primary pancreatic resection. J. Gastrointest. Surg..

[21] Shimoike N., Fujikawa T., Maekawa H., Tanaka A. (2013). Aggressive secondary surgery for local recurrence of pancreatic cancer. BMJ Case Rep.

[22] Akabori H., Shiomi H., Naka S., Murakami K., Murata S., Ishida M., Kurumi Y., Tani T. (2014). Resectable carcinoma developing in the remnant pancreas 7 years and 10 months after distal pancreatectomy for invasive ductal carcinoma of the pancreas: Report of a case. World J. Surg. Oncol..

[23] Boone B.A., Zeh H.J., Mock B.K., Johnson P.J., Dvorchik I., Lee K., Moser A.J., Bartlett D.L., Marsh J.W. (2014). Resection of isolated local and metastatic recurrence in periampullary adenocarcinoma. HPB.

[24] Hashimoto D., Chikamoto A., Ohmuraya M., Sakata K., Miyake K., Kuroki H., Watanabe M., Beppu T., Hirota M., Baba H. (2014). Pancreatic cancer in the remnant pancreas following primary pancreatic resection. Surg. Today.

[25] Miyazaki M., Yoshitomi H., Shimizu H., Ohtsuka M., Yoshidome H., Furukawa K., Takayasiki T., Kuboki S., Okamura D., Suzuki D. (2014). Repeat pancreatectomy for pancreatic ductal cancer recurrence in the remnant pancreas after initial pancreatectomy: Is it worthwhile?. Surgery.

[26] Sunagawa H., Mayama Y., Orokawa T., Oshiro N. (2014). Laparoscopic total remnant pancreatectomy after laparoscopic pancreaticoduodenectomy. Asian J. Endosc. Surg..

[27] Balaj C., Ayav A., Oliver A., Jausset F., Sellal C., Claudon M., Laurent V. (2016). Ct imaging of early local recurrence of pancreatic adenocarcinoma following pancreaticoduodenectomy. Abdom. Radiol..

[28] Hardacre J.M. (2016). Completion pancreaticoduodenectomy for a second primary pancreatic cancer: A case report. Case Rep. Pancreat. Cancer.

[29] Ishida J., Toyama H., Matsumoto I., Asari S., Goto T., Terai S., Nanno Y., Yamashita A., Mizumoto T., Ueda Y. (2016). Second primary pancreatic ductal carcinoma in the remnant pancreas after pancreatectomy for pancreatic ductal carcinoma: High cumulative incidence rates at 5 years after pancreatectomy. Pancreatology.

[30] Sahakyan M.A., Yaqub S., Kazaryan A.M., Villanger O., Berstad A.E., Labori K.J., Edwin B., Røsok B.I. (2016). Laparoscopic completion pancreatectomy for local recurrence in the pancreatic remnant after pancreaticoduodenectomy: Case reports and review of the literature. J. Gastrointest. Cancer.

[31] Suzuki S., Furukawa T., Oshima N., Izumo W., Shimizu K., Yamamoto M. (2016). Original scientific reports: Clinicopathological findings of remnant pancreatic cancers in survivors following curative resections of pancreatic cancers. World J. Surg..

[32] Zhou Y., Song A., Wu L., Si X., Li Y. (2016). Second pancreatectomy for recurrent pancreatic ductal adenocarcinoma in the remnant pancreas: A pooled analysis. Pancreatology.

[33] Groot V.P., van Santvoort H.C., Rombouts S.J., Hagendoorn J., Borel Rinkes I.H., van Vulpen M., Herman J.M., Wolfgang C.L., Besselink M.G., Molenaar I.Q. (2017). Systematic review on the treatment of isolated local recurrence of pancreatic cancer after surgery; re-resection, chemoradiotherapy and sbrt. HPB.

[34] Kim N.H., Kim H.J. (2018). Preoperative risk factors for early recurrence in patients with resectable pancreatic ductal adenocarcinoma after curative intent surgical resection. Hepatobiliary Pancreat. Dis. Int..

[35] Nakayama Y., Sugimoto M., Gotohda N., Konishi M., Takahashi S. (2018). Efficacy of completion pancreatectomy for recurrence of adenocarcinoma in the remnant pancreas. J. Surg. Res..

[36] Kim Y.I., Song K.B., Lee Y.J., Park K.M., Hwang D.W., Lee J.H., Shin S.H., Kwon J.W., Ro J.S., Kim S.C. (2019). Management of isolated recurrence after surgery for pancreatic adenocarcinoma. Br. J. Surg..

[37] Moletta L., Serafini S., Valmasoni M., Pierobon E.S., Ponzoni A., Sperti C. (2019). Surgery for recurrent pancreatic cancer: Is it effective?. Cancers.

[38] Suzuki S., Shimoda M., Shimazaki J., Maruyama T., Nishida K. (2019). Clinical outcome of resected remnant pancreatic cancer after resection of the primary pancreatic cancer. J. Investig. Surg..

[39] Shima Y., Okabayashi T., Kozuki A., Sumiyoshi T., Tokumaru T., Saisaka Y., Date K., Iwata J. (2015). Completion pancreatectomy for recurrent pancreatic cancer in the remnant pancreas: Report of six cases and a review of the literature. Langenbeck Arch. Surg..

[40] Rahib L., Smith B.D., Aizenberg R., Rosenzweig A.B., Fleshman J.M., Matrisian L.M. (2014). Projecting cancer incidence and deaths to 2030: The unexpected burden of thyroid, liver, and pancreas cancers in the united states. Cancer Res..

[41] Katz M.H., Pisters P.W., Evans D.B., Sun C.C., Lee J.E., Fleming J.B., Vauthey J.N., Abdalla E.K., Crane C.H., Wolff R.A. (2008). Borderline resectable pancreatic cancer: The importance of this emerging stage of disease. J. Am. Coll. Surg..

[42] Tuveson D.A., Neoptolemos J.P. (2012). Understanding metastasis in pancreatic cancer: A call for new clinical approaches. Cell.

[43] Haeno H., Gonen M., Davis M.B., Herman J.M., Iacobuzio-Donahue C.A., Michor F. (2012). Computational modeling of pancreatic cancer reveals kinetics of metastasis suggesting optimum treatment strategies. Cell.

[44] Jones R.P., Psarelli E.E., Jackson R., Ghaneh P., Halloran C.M., Palmer D.H., Campbell F., Valle J.W., Faluyi O., O’Reilly D.A. (2019). Patterns of recurrence after resection of pancreatic ductal adenocarcinoma: A secondary analysis of the espac-4 randomized adjuvant chemotherapy trial. JAMA Surg..

[45] Fortner J.G. (1981). Surgical principles for pancreatic cancer: Regional total and subtotal pancreatectomy. Cancer.

[46] Choi M., Lee S.J., Shin D.M., Hwang H.K., Lee W.J., Kang C.M. (2020). Laparoscopic repeated pancreatectomy for isolated local recurrence in remnant pancreas following laparoscopic radical pancreatectomy for pancreatic ductal adenocarcinoma: Two cases report. Ann. Hepatobiliary Pancreat. Surg..

